# Cutaneous Rosai-Dorfman disease with near-complete response to the MEK inhibitor cobimetinib

**DOI:** 10.1016/j.jdcr.2025.07.003

**Published:** 2025-07-17

**Authors:** Vyshnavi Rallapalle, Lauren Kole, Peter Pavlidakey, Pradeep Bhambhvani, Shuko Harada, Aishwarya Ravindran, Gaurav Goyal

**Affiliations:** a Heersink School of Medicine, University of Alabama at Birmingham, Birmingham, Alabama; b Department of Dermatology, University of Alabama at Birmingham, Birmingham, Alabama; c Department of Radiology, University of Alabama at Birmingham, Birmingham, Alabama; d Division of Genomic Diagnostics and Bioinformatics, Department of Pathology, University of Alabama at Birmingham, Birmingham, Alabama; e Division of Laboratory Medicine-Hematopathology, Department of Pathology, University of Alabama at Birmingham, Birmingham, Alabama; f Division of Hematology-Medical Oncology, University of Alabama at Birmingham, Birmingham, Alabama

**Keywords:** cobimetinib, cutaneous, histiocytosis, MAPK/ERK pathway, MEK inhibitor, oncology, pharmacology, Rosai-Dorfman disease

## Introduction

Rosai-Dorfman disease (RDD) is a rare histiocytic disorder that can affect any organ system of the body, most commonly involving the skin and lymph nodes.^1^ Histopathologic features are characterized by accumulation of enlarged histiocytes with frequent emperipolesis (engulfment of inflammatory cells) and positive expression of macrophage markers (CD68 and CD163), S100, OCT2, and negative expression of dendritic/Langerhans cell markers (CD1a and langerin).^2^ RDD was previously considered an inflammatory condition, but the discovery of oncogenic mitogen-activated protein kinase/extracellular signal-regulated kinase (MAPK/ERK) pathway mutations in nearly 40% of cases has led to its recognition as a neoplastic disorder by the World Health Organization of hematopoietic tumors in 2022.^3^ However, many patients may still not get appropriate staging and referral to hematology-oncology due to a lack of awareness about this condition.

The clinical course of cutaneous RDD is presumed to be benign, with many cases resolving spontaneously or with surgical excision or topical therapies.^4^ Recently, cobimetinib, an oral mitogen-activated extracellular signal-regulated kinase (MEK) inhibitor was approved by the US Food and Drug Administration for histiocytic neoplasms, including RDD, due to excellent response rates in a phase 2 study.^5^ However, the role of cobimetinib in cutaneous-only RDD is unclear, as most patients tend to receive skin-directed treatments or surgery. We present a rare case of a large cutaneous RDD lesion that progressed despite initial topical therapy and had a remarkable response to treatment with cobimetinib.

## Case report

**Fig 1:**
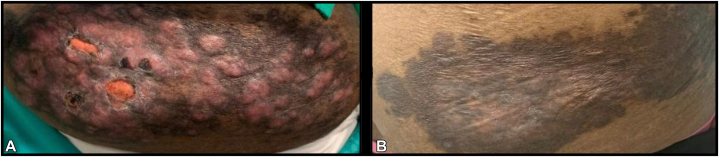
**A** and **B,** Cutaneous Rosai-Dorfman disease: Clinical photographs. **A,** At diagnosis: Initial presentation of a round, erythematous nodular plaque on the right buttock measuring ∼20 centimeters in greatest dimension. **B,** Post-treatment: Clinical response demonstrating a marked improvement with flattening and lightening of the skin lesion at 3 months post-cobimetinib therapy.

**Fig 2:**
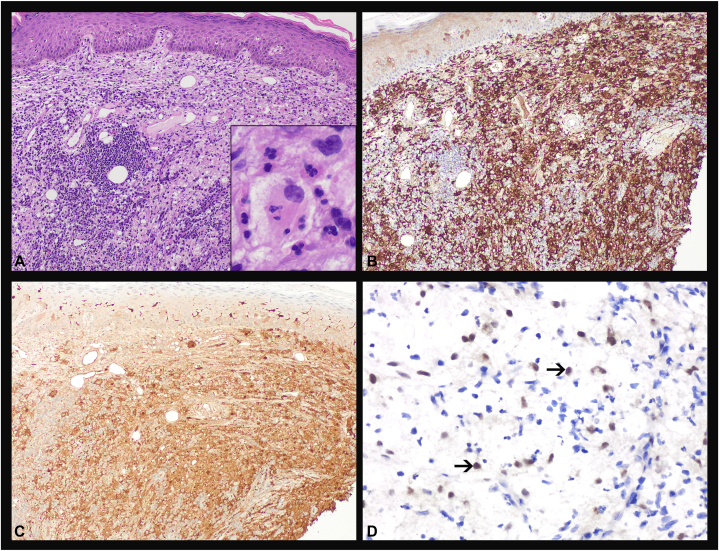
**A-D**, Cutaneous Rosai-Dorfman disease: Histopathological features. **A,** Light microscopy (hematoxylin and eosin stain, ×100 [inset ×400]) demonstrates a portion of skin biopsy with a diffuse dermal involvement by abnormal histiocytic infiltrates that are characterized by enlarged *round to oval* nuclei, conspicuous nucleoli, pale chromatin, and abundant pale eosinophilic cytoplasm with frequent engulfment of inflammatory cells composed of predominantly lymphocytes, plasma cells, and neutrophils (emperipolesis, inset). By immunohistochemistry, these atypical histiocytes are diffusely positive for CD163 (**B,** ×100), S100 (**C,** ×100), and cyclin D1 (**D,** ×400; *black arrows* indicate RDD histiocytes). *RDD*, Rosai-Dorfman disease.

A 76-year-old Black female with a past medical history of bilateral invasive mammary carcinoma presented to the dermatology clinic for evaluation of an itchy, progressively enlarging rash on her back and right buttock. On physical examination, the patient had a well-demarcated, round, erythematous, nodular plaque with hyperpigmented borders on her right buttock measuring approximately 20 centimeters in greatest dimension (Fig 1, *A*), and a similar smaller lesion present on her left mid-back. A punch biopsy of the lesion on her buttock revealed histopathologic findings diagnostic of RDD with positive immunostaining for CD163, S100, and cyclin D1 (Fig 2, *A-D*).

Initial treatment involved topical triamcinolone, betamethasone, and intralesional triamcinolone injections, with the lesions showing mild improvement but eventually becoming larger and more eroded, causing pruritus. She was referred to hematology-oncology at this point for further management. She underwent an 18F-fluorodeoxyglucose positron emission tomography scan showing right buttock and left back hypermetabolic nodules correlating with the physical exam without any internal organ involvement. Commercially available next-generation sequencing (NGS) was pursued on the skin biopsy specimen, and no pathogenic variants or fusion transcripts in the MAPK/ERK pathway were reported.

**Fig 3:**
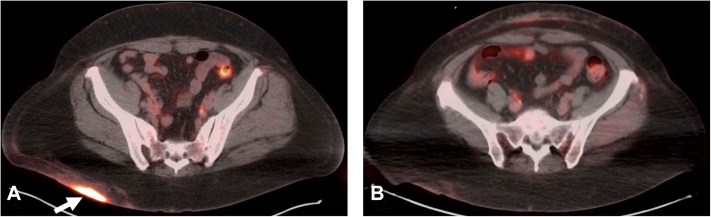
**A** and **B,** Cutaneous Rosai-Dorfman disease: 18-fluorodeoxyglucose-positron emission tomography (FDG-PET) images. **A,** At diagnosis: Initial presentation of hypermetabolic lesion on the right buttock on FDG-PET scan (indicated by *white arrow*). **B,** Post-treatment: FDG-PET scan images demonstrating complete resolution of the lesion at 9 months post-cobimetinib therapy.

Due to the persistence of symptoms and given the recent Food and Drug Administration approval of MEK inhibitors for adult histiocytosis,^5^^,^^6^ the patient was treated with 40 mg cobimetinib orally from days 1-21 of 28-day cycles (1 week break every month). This led to rapid clinical improvement with the lesions flattening and lightening at her 3-month follow-up appointment (Fig 1, *B*). After 9 months of therapy, the patient had continued improvement in the size of her lesions with almost complete resolution of the erythematous aspects and pruritus and complete resolution of her hypermetabolic nodules on her 18F-fluorodeoxyglucose positron emission tomography scan (Fig 3). She had no major side effects from her oral therapy except for mild fatigue. Retrospectively, the hot spots of key genes involving the MAPK/ERK pathway were reviewed in the raw data from NGS, identifying a *MAP2K1* p.F53I pathogenic mutation at a variant allelic frequency of 4.6%. Since most commercially available NGS only report pathogenic variants at variant allelic frequency ≥5%, this was not initially reported.

## Discussion

Our case highlights the role of a single-agent targeted MEK inhibitor, cobimetinib, in the treatment of cutaneous RDD, especially when skin-directed treatments do not succeed. Through its inhibition of MEK1/2, cobimetinib targets a key part of the MAPK/ERK pathway. A recent study evaluated the efficacy of cobimetinib in RDD in 16 patients, but only 1 patient had isolated skin disease with stability of their lesion at 10-month follow-up.^6^ This study found that the response to cobimetinib for those with MEK or KRAS mutations was much higher compared to those without (88% vs 38%).^6^ Another reported case found that a topical MEK inhibitor, trametinib, was effective in treating cutaneous RDD when combined with oral methotrexate and intralesional triamcinolone injections applied directly to the lesions.^7^ While patients with multisystem disease or internal organ involvement by RDD often are referred to hematology-oncology, ones with cutaneous-only disease may be managed by dermatology alone.

There is currently no evidence-based standard of care treatment for cutaneous RDD due to the rarity of the disease. A recent systematic review found that the most commonly used therapy for cutaneous RDD involved surgical excision, with complete response achieved in 94% of cases.^4^ The most common second-line treatments included systemic corticosteroids and topical corticosteroids, with systemic therapy being much more effective than topical treatment.^4^ Other treatment options included retinoids, thalidomide, methotrexate, chemotherapy, and observation, all with variable results.^4^

This case underscores an avenue for targeted therapy for treatment-refractory cases of RDD. Reasons for hesitancy to use cobimetinib include the broad side effect profile of MEK inhibitor monotherapy. Common side effects include photosensitivity reactions, papulopustular rashes, hepatotoxicity, fatigue, and ocular toxicities.^8^^,^^9^ Our patient suffered from grade 1 fatigue, but her symptoms resolved during the days off of her therapy, allowing her to continue treatment.

It is vital for patients with cutaneous RDD to be referred to hematology-oncology for complete staging and consideration of targeted therapies, especially if focal dermatologic treatments are unsuccessful. While this report is promising, additional challenges remain in the management of RDD using targeted inhibitors, especially the risk of disease recurrence after stopping the drug, which necessitates prolonged administration of treatment. Future studies are needed to investigate the optimum duration of treatment and other limited-duration treatments that are curative.

## Conflicts of interest

Dr Goyal has served on the advisory board for Seagen and Sobi and received consulting fees from Recordati and Pharmassentia. Drs Rallapalle, Kole, Pavlidakey, Bhambhvani, Harada, and Ravindran have no conflicts of interest to declare.
